# Diffusion Tensor Imaging Biomarkers to Predict Neurological Outcomes in Brain Surgery: A Systematic Review

**DOI:** 10.3390/life16010115

**Published:** 2026-01-13

**Authors:** Noa Ben Dor, Giovanni Sighinolfi, Vittoria Rosetti, Filippo Friso, Giada Garufi, Salvatore Massimiliano Cardali, Caterina Tonon, Raffaele Lodi, Alfredo Conti

**Affiliations:** 1 Department of Neurosurgery, IRCCS Institute of Neurological Sciences of Bologna, 40139 Bologna, Italy; filippo.friso@studio.unibo.it (F.F.); raffaele.lodi@unibo.it (R.L.); alfredo.conti2@unibo.it (A.C.); 2 Department of Biomedical and Neuromotor Sciences (DIBINEM), University of Bologna, 40138 Bologna, Italy; vittoria.rosetti2@unibo.it (V.R.); caterina.tonon@unibo.it (C.T.); 3 Functional and Molecular Neuroimaging Unit, IRCCS Institute of Neurological Sciences of Bologna, 40139 Bologna, Italy; giovanni.sighinolfi3@unibo.it; 4 Department of Neurosurgery, Azienda Ospedaliera Papardo, University of Messina, 98158 Messina, Italy; giadagarufi@hotmail.it (G.G.);

**Keywords:** neurosurgery, diffusion tensor imaging (DTI), fiber tracking, fractional anisotropy (FA), corticospinal tract (CST), surgical outcome

## Abstract

Diffusion tensor imaging (DTI) tractography is routinely employed in neurosurgical planning; however, the prognostic significance of quantitative DTI metrics for postoperative functional outcomes remains unclear. We conducted a PRISMA-informed systematic review of PubMed (January 2005–1 December 2025), supplemented by additional indexed sources, to synthesize the evidence on quantitative DTI measures associated with postoperative motor, language, and cognitive outcomes following intracranial surgery. Thirty-seven studies were included, primarily single-center studies, and predominantly focused on glioma surgery. Motor outcomes exhibited the most consistent associations, with reduced corticospinal tract integrity and adverse postoperative diffusion changes correlating with muscle weakness and poorer recovery. Recovery from supplementary motor area syndrome was associated with interhemispheric callosal connectivity, with greater disconnection predicting a prolonged symptom duration. Language outcomes demonstrated reproducible structure–function relationships: higher preoperative integrity of the dorsal language pathways was associated with milder postoperative aphasia and better recovery, whereas postoperative tract disruption and diffusivity changes predicted persistent naming and fluency deficits, and ventral pathway alterations were specifically linked to lexico-semantic impairment. In epilepsy surgery, language performance correlated with contralateral and distributed network diffusion changes, consistent with reorganization. Evidence for cognition and gait outcomes was limited and mainly involved the association, limbic, and callosal pathways. Overall, quantitative DTI provides clinically relevant markers of tract and network disruption and postoperative remodeling; however, methodological heterogeneity and limited external validation currently preclude universal prognostic thresholds.

## 1. Introduction

Diffusion magnetic resonance imaging (dMRI) has become an integral part of preoperative planning for intracranial surgery, particularly in patients with lesions close to eloquent white matter pathways [1]. In this setting, dMRI is especially useful, as proper sequences enable tractography, that is, the computational reconstruction of white matter fiber pathways [2]. This capability is crucial in neuro-oncology, where the goal is to maximize lesion removal while preserving the subcortical pathways that support motor, language, and cognitive functions [3]. However, tractography is only one of several dMRI applications in this field. Another promising application is the investigation of apparently normal, non-pathological tissue microstructures. While several models have been developed over the years, diffusion tensor imaging (DTI) is the most commonly used in the scientific community with clinical applications, particularly because of the relative simplicity of the model and the complexity of the data required to perform this processing, in addition to historical reasons [4,5].

**Figure 1:**
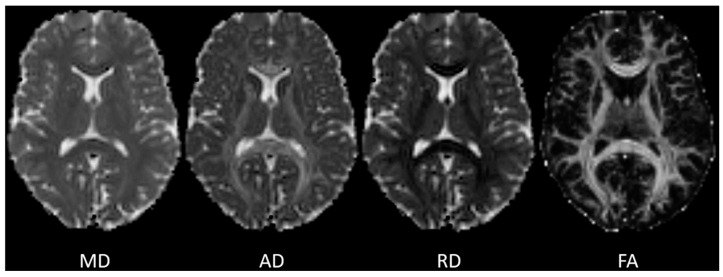
Example of whole-brain voxel-wise maps of diffusion tensor imaging metrics. MD, mean diffusivity; AD, axial diffusivity; RD, radial diffusivity; FA, fractional anisotropy.

DTI relies on modeling water diffusion in each voxel as a tensor, that is, a 3 × 3 matrix decomposition of the diffusion coefficient along the three spatial axes, rather than a single scalar value. This matrix can be mathematically represented by an ellipsoid. The major and minor axes of the diffusion ellipsoid are defined by three orthogonal unit vectors (εi), known as eigenvectors, which describe the main directions of diffusion, and their lengths (λi), called eigenvalues, which are related to the diffusivity value in that direction [6]. Based on this framework, several scalar metrics interpreted as tissue microstructural characteristics can be derived. The DTI model defines a set of scalar quantities that locally describe the diffusion properties of the tissue based on the eigenvalues λi. The first measure is the mean diffusivity (MD), which is the average eigenvalue. MD is related to the total amount of diffusion in a voxel, which is inversely related to the amount of water in the extracellular space. Connected measures are the axial (AD, equal to λ1) and radial (RD, equal to the average of λ2 and λ3) diffusivities, which describe the diffusivity along the main direction of diffusion and in the orthogonal plane, respectively. The second commonly used metric is the fractional anisotropy (FA), which measures the fraction of diffusion that is anisotropic (i.e., the existence of one preferential direction of diffusion opposed to the other axes) and can be interpreted as the difference between the diffusion ellipsoid and the perfect sphere along a single direction. An example of the voxel-wise parameter maps of MD, AD, RD, and FA is shown in Figure 1.

Additionally, microstructural modelling and tractography can be combined to perform along-tract analysis, that is, the investigation of voxel-wise parameter maps (such as FA and MD) along the course of a specific, tractography-reconstructed tract, rather than calculating the statistic over un-specific regions of interest [7].

Alterations in DTI metrics can result from axonal injury, demyelination, edema, increased cellularity, or tract displacement, making them sensitive, albeit not specific, indicators of microstructural changes [6]. Both experimental and clinical studies indicate that a decrease in FA, along with an increase in MD or RD, generally indicates axonal disorganization, edema, or demyelination rather than complete axonal loss [8]. However, interpreting these changes is complex, particularly when considered in isolation and without strong a priori hypotheses regarding the potential neurophysiological origins of the alterations, as they may be attributed to various factors [9].

In neurosurgical patients, tumors, cavernomas, hemorrhages, and hydrocephalus may infiltrate, compress, or disconnect key pathways, such as the corticospinal tract (CST), language-related association fibers, and commissural connections. Qualitative tractography is widely used to map these tracts; however, quantitative DTI provides additional information on the microstructural burden and residual “reserve” of the affected pathways [10]. In the stroke literature, early reductions in CST FA and related metrics have been associated with poorer motor recovery, and DTI-based biomarkers have been evaluated as predictors of rehabilitation potential [11]. A similar prognostic role for DTI metrics has been suggested in neurosurgical cohorts; however, the evidence is scattered across small, heterogeneous studies and has not yet been comprehensively synthesized.

Understanding whether and how DTI metrics predict functional preservation and recovery after brain surgery is clinically relevant for several reasons. First, robust preoperative markers of tract vulnerability and neuroplastic potential could refine risk stratification, inform patient counseling, and guide the extent of resection and approach [12]. Second, early postoperative DTI may help differentiate transient deficits due to edema or temporary disconnection from permanent injury, thereby influencing prognosis and rehabilitation planning [13]. Third, longitudinal DTI can serve as an imaging biomarker for structural reorganization and treatment responses [14].

Therefore, this systematic review aimed to achieve the following objectives.

1. The following summarizes the use of quantitative DTI metrics to characterize lesions and surgery-related white matter changes in patients undergoing brain surgery.
2. To evaluate the evidence for DTI based prediction of motor, language, gait and cognitive outcomes across neurosurgical conditions; and
3. To identify the methodological limitations and priorities for future research needed to establish DTI as a reliable prognostic biomarker in neurosurgical practice.

## 2. Materials and Methods

### 2.1. Study Design and Search Strategy

To ensure a robust and transparent approach to our literature search and analysis, this systematic review was designed in accordance with the PRISMA guidelines [15]. The search was conducted in MEDLINE (Medical Literature Analysis and Retrieval System Online; through the PubMed interface) from January 2005 to August 2025, using the following keywords: Diffusion Tensor Imaging (or diffusion tensor, DTI, tractography, fiber tracking) and functional outcome (or motor, motor recovery, language, aphasia, paresis, cortical reorganization, plasticity) and surgery (or resection, postoperative, craniotomy). The full PubMed search string is provided in Appendix A. To reduce publication bias and identify additional eligible studies, we queried the Elicit platform (Semantic Scholar and OpenAlex) [16] and screened the reference lists of the included articles and relevant reviews to identify additional studies.

Studies were considered eligible if they involved adult patients who underwent intracranial surgery, such as tumor resection, epilepsy surgery, hematoma evacuation, or shunt placement. Methodological descriptions are required to provide comprehensive details on diffusion MRI acquisition, DTI processing methods (including preprocessing steps, tensor or higher-order modeling, and software used), and post-processing strategies (whole-brain, region-of-interest, or tractography). Studies were included only if they evaluated at least one DTI metric (FA, AD, RD, or MD), while those performing tractography without reporting DTI metrics were excluded. Additionally, inclusion necessitated the reporting of quantifiable postoperative functional outcomes (motor, language, and cognitive) assessed at least once beyond the immediate perioperative period. We deliberately concentrated on synthesizing DTI-derived quantitative metrics (FA, MD/ADC, AD, RD) because they are the most frequently reported diffusion biomarkers in neurosurgical outcome studies and are widely used in clinical practice. Studies that utilized diffusion acquisitions but did not report tensor metrics (e.g., those focusing solely on qualitative tractography) were excluded from the study. Eligible designs encompassed original clinical investigations, including prospective or retrospective cohort studies, case series, and single-case reports, with full-text articles available in English. Reviews, meta-analyses, editorials, technical notes without patient data, and conference abstracts were excluded from the study.

### 2.2. Study Selection and Data Extraction

All identified citations were imported into a reference management platform (Zotero version 7.x; open-source software; https://www.zotero.org). After removing duplicates, the first author screened the titles and abstracts to exclude records that were clearly unrelated to neurosurgery, did not perform quantitative diffusion MRI analysis, or did not report postoperative clinical data. Full texts were retrieved for studies deemed potentially relevant, and their eligibility was assessed in detail against the predefined criteria. The second author examined the equivocal cases and verified the final set of included studies.

The following information was retrieved from the manuscripts retained for the qualitative synthesis: study design and sample size, patient population and neurosurgical procedure, lesion type and location, regions of interest (ROIs) or tracts studied, quantitative DTI metrics, timing of imaging (preoperative, early postoperative, longitudinal follow-up), neurological outcomes (motor, language, cognition), and main reported associations between DTI metrics and clinical outcomes. For studies reporting cognitive and/or language outcomes, we also extracted the neuropsychological tests/batteries and assessment timepoints, summarized in Supplementary Table S1.

A meta-analysis was not performed because the included studies were not sufficiently comparable for valid quantitative pooling. Heterogeneity was present in: (i) outcome definitions and measurement scales (e.g., transient vs. persistent deficits; bedside grades vs. standardized batteries), (ii) follow-up timing (discharge, weeks, months, ≥1 year), (iii) diffusion acquisition and preprocessing (field strength, gradient directions, b-values, distortion/eddy correction), (iv) tract definition and tractography methodology (deterministic vs. probabilistic tracking, ROI placement, FA thresholds, atlas-based vs. subject-specific reconstruction), and (v) reporting formats (correlations, odds ratios, AUCs, or thresholds; frequent missing variance estimates and inconsistent covariate adjustment). Given these differences and incomplete reporting of harmonizable effect sizes, quantitative synthesis risked producing misleading pooled estimates. Therefore, we performed a structured qualitative synthesis grouped by clinical domain (motor, language, cognition, and gait). A detailed discussion of the heterogeneity drivers is provided in Section 4.3.

## 3. Results

### 3.1. Search Results

**Figure 2:**
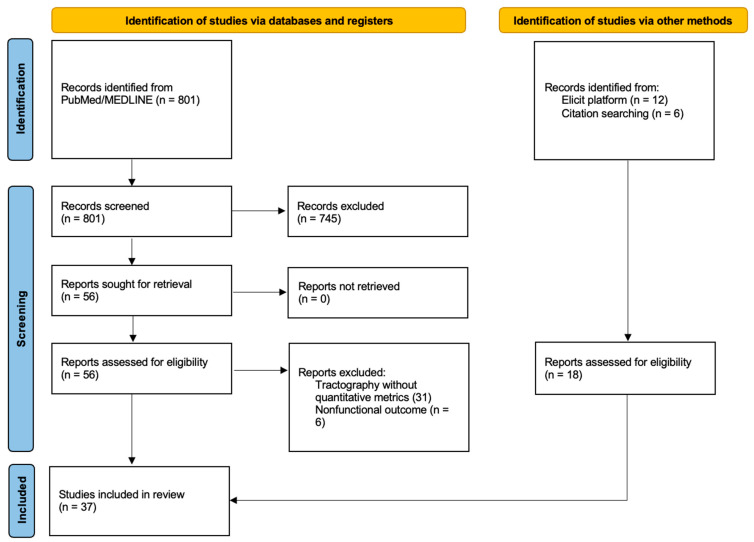
PRISMA flow diagram of the literature search and selection of relevant studies.

The PubMed search identified 801 records published between 2005 and 2025. After title/abstract screening, 745 records were excluded, and 56 full texts were assessed for their eligibility. Nineteen studies met the inclusion criteria [17,18,19,20,21,22,23,24,25,26,27,28,29,30,31,32,33,34,35] and were included in this systematic review. Twelve additional eligible studies were identified via the Elicit platform [33,36,37,38,39,40,41,42,43,44,45,46] and six via reference screening [47,48,49,50,51,52], yielding 37 studies included in the qualitative synthesis (Figure 2).

### 3.2. Characteristics of Included Studies

**Figure 3:**
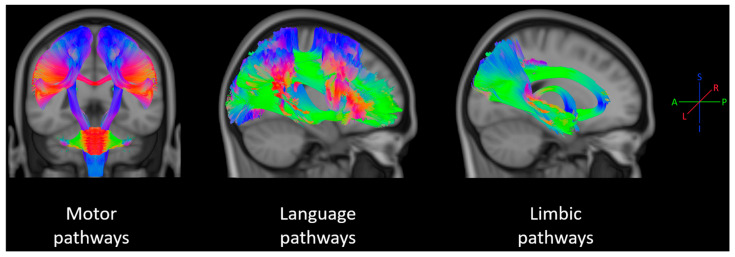
Representation of the tractography-reconstructed main tracts supporting motor, language, and cognitive (within the limbic system) functions. The represented motor pathways included the cortico-spinal tract, cerebellar peduncles, frontal aslant tract, and motor corpus callosum; language pathways included the arcuate fasciculus, superior longitudinal fasciculus, inferior fronto-occipital fasciculus, uncinate fasciculus, inferior longitudinal fasciculus, frontal aslant tract; and limbic pathways included the cingulum bundle, fornix, and middle temporal tract. Template tracts were selected from the KUL-FWT atlas [55]. The color encoding follows the typical tractography representation [56,57]: red for right-left streamlines, green for anterior-posterior, and blue for inferior-superior.

Table 1 summarizes the key characteristics of the 37 studies included in this review. Most studies examined patients undergoing glioma resection [18,20,21,22,25,27,29,35,36,44,45,47,51,53], with additional studies investigating epilepsy surgery [39,46] and other conditions, including brainstem cavernoma [52], basal ganglia hemorrhage [42], moyamoya disease [40], and normal pressure hydrocephalus [38]. The sample sizes ranged from 5 to 360 patients, with the majority being single-center retrospective or prospective cohort studies. The primary outcome domains included motor function [20,21,22,23,24,25,26,27,28,33,35,36,41,42,44,48,51,52], language function [17,18,19,30,37,39,43,45,46,49,54], and broader cognitive assessments [37,38,40] (Figure 3). 

### 3.3. DTI Metrics and Technical Approaches

Diffusion imaging was performed on 1.5 T or 3 T scanners using single-shot echo-planar sequences. The number of diffusion-encoding directions ranged from 6 to 64, with b values typically between 800 and 1000 s/mm^2^ and at least one b0 image. Preprocessing pipelines included correction for eddy currents and susceptibility-induced distortions, brain extraction, and tensor estimation. Most studies have utilized deterministic tractography algorithms with FA thresholds ranging from 0.1 to 0.2. The analysis software included FSL [17,18,19,24,40,45,46], Brainlab Elements [20,29,37], and DSI Studio [20,21,27].

ROIs were defined manually in many studies, semi-automatically using atlases in others, or post hoc from significant clusters in voxel wise analyses. The white matter pathways of interest, which are visually represented in Figure 2, included the following:

- Motor pathways: CST from the precentral gyrus through the corona radiata, posterior limb of the internal capsule, cerebral peduncle, pons, and medulla; frontal aslant tract (FAT); premotor and SMA connections, and corpus callosum segments connecting right and left SMAs [20,21,22,23,24,25,26,27,28,34,36,41,42,44,47,48,50,52,53].
- Language pathways: arcuate fasciculus (AF) and its long and segmental components, superior longitudinal fasciculus (SLF), inferior fronto-occipital fasciculus (IFOF), uncinate fasciculus, inferior longitudinal fasciculus (ILF), frontal aslant tract (FAT) [17,19,35,39,43,45,46,49,54].
- Limbic and medial temporal pathways: fornix, cingulum bundle, and medial temporal subnetworks [37,38,40].

The quantitative measures reported fall into three broad categories:

- Voxel-wise tensor metrics: FA in the majority of studies; MD, AD, and RD in a subset, particularly in temporal lobe epilepsy, glioma, and brainstem cavernoma cohorts [18,20,21,23,24,25,26,27,30,31,32,33,36,37,38,39,40,41,42,43,44,45,46,47,48,49,50,51].
- Tract-based tensor metrics: mean FA and diffusivity along a reconstructed tract, and laterality indices for FA and MD (e.g., ipsilateral vs. contralateral CST) [19,22,24,28,35].
- Additional tract-based indices: streamline count, tract length, volume, and measures of callosal or interhemispheric disconnection based on disconnectome mapping [17,34,39,54,58].

### 3.4. Imaging Timepoints

Preoperative DTI was performed in all the studies. Early postoperative imaging, ranging from postoperative day 1 to approximately 6 weeks, is generally used to relate acute tract changes to new neurological deficits [20,23,42,43,46]. Longitudinal follow-up spanned from 3 months to over one year, particularly in glioma and temporal lobe epilepsy populations, and in iNPH cohorts assessed before and after shunting [38,40,45].

### 3.5. Predictive Value for Motor Function Outcomes

Motor outcomes represented the largest evidence base across the included studies. DTI-based approaches predominantly quantified corticospinal tract (CST) integrity using fractional anisotropy (FA) (often expressed as a side-to-side ratio), diffusivity measures (ADC/MD, RD, AD), and—in a smaller subset—DTI tractography–derived indices such as fiber/streamline-count metrics (e.g., NF index, NFidx) and tract “disconnection severity”. These measures were then related to (i) baseline motor status, (ii) early postoperative motor deficits and their persistence, or (iii) recovery trajectories, including recovery from supplementary motor area (SMA) syndrome.

#### 3.5.1. Preoperative DTI-Derived CST Integrity Predicting Postoperative Motor Deficits

In a retrospective analysis by Ivren et al. (2023), a study of 203 patients with motor-eloquent gliomas examined the postoperative motor outcomes [25]. Postoperatively, 57.1% of patients had no new deficits, 24.1% developed transient deficits, and 18.8% developed permanent deficits. Their diffusion analysis showed that lower FA values were linked to higher postoperative motor deterioration, with FA values associated with new transient (OR 3.0, 95% CI 1.5–6.0) and permanent (OR 7.4, 95% CI 2.8–19.5) deficits. Their predictive model reported an AUC of 0.79 [25]. Ius et al. (2017) studied 37 LGG patients and introduced the NF index, calculated as (Hcst NF—Tcst NF)/Hcst NF, where NF represents CST fibers in healthy (H) and tumoral (T) hemispheres [48]. In the ROC analysis, the NF index identified temporary postoperative motor deficits with an AUC of 0.92 (95% CI, 0.834–1.000). An NF index of <0.22 indicated an 87.5% probability of no early deficit, while ≥0.22 indicated an 81% probability of deficit. Compared to MEPs, the NF index showed fewer false negatives (13.3%) and high reliability (86.5%). The authors noted that tractography results depend on acquisition choices and should complement other decision tools [48].

#### 3.5.2. Early Postoperative DTI Changes and Longitudinal Monitoring of Motor Recovery

Cepeda et al. (2021) investigated 11 patients with glioma and related early postoperative DTI changes to motor outcomes [20]. They quantified CST/peritumoral DTI metrics bilaterally, focusing on the relative FA (rFA) (ipsilesional/contralesional). In group comparisons, rFA differed across outcome groups (Kruskal–Wallis H = 7.48, *p* = 0.024, effect size ε^2^ = 0.75), with permanent deficit patients showing the lowest rFA values. For permanent deficit classification, all three patients with permanent motor deficit had rFA ≤ 0.8, achieving 100% sensitivity and 75% specificity. The ROC curve showed an AUC of 0.917. Cepeda et al. concluded that early postoperative DTI, especially rFA, marks persistent motor deficits, noting that postoperative DTI changes reflect surgical effects and microstructural alterations [20].

Figueredo et al. (2024) presented two illustrative cases involving the resection of gliomas in the primary motor area, with CST FA measurements taken preoperatively, immediately postoperatively, and at a 1-year follow-up [21]. In Case 1, the FA values were 0.50, 0.47, and 0.42, whereas in Case 2, the values were 0.49, 0.44, and 0.44. The authors noted that, within this limited sample, changes in DTI-derived parameters, including FA, were not statistically significant at different time points. They positioned the report as a feasibility study and explicitly acknowledged the limitation of a very small sample size, which restricted inference and precluded robust statistical conclusions [21].

Horikawa et al. (2025) presented a detailed case study with serial DTI and functional assessments [23]. They reported that rFA declined below 0.8 on postoperative day 12 and subsequently increased above 0.8 on days 29 and 134, paralleling clinical recovery in strength and dexterity, as documented in their longitudinal assessment. The authors’ conclusions were explicitly limited by the single-patient design and presented primarily as descriptive evidence supporting the need for continued rehabilitation [23].

Liao et al. (2025) compared 104 patients with minor basal ganglia hemorrhage managed with stereotactic surgery plus medical therapy (n = 52) versus medical therapy alone (n = 52) [42]. The DTI indices (FA, MD, RD, and AD) were sampled at the posterior limb of the internal capsule at 48 h and 1 month, alongside motor impairment scores (Fugl–Meyer motor function) and 6 month disability (mRS). At 1 month, FA was higher in the surgery group (0.56 vs. 0.51, *p* < 0.001) and RD was lower (0.42 vs. 0.49, *p* < 0.001); MD was also lower (0.79 vs. 0.86, *p* < 0.001), while AD did not differ significantly. Clinically, at 6 months, the distribution of disability differed between groups, with mRS 0–1 in 75.0% of the surgery group versus 42.3% of the medical group (*p* < 0.001) [42].

Non-tumor studies have also enriched the evidence-based literature on this topic. In the context of brainstem cavernoma surgery, Yao et al. (2015) identified quantitative correlations between corticospinal tract (CST) integrity and motor deficits in patients [52]. Specifically, the preoperative mean fractional anisotropy (FA) was correlated with preoperative motor deficits (Spearman’s ρ = 0.63, *p* = 0.014) and postoperative motor outcomes (ρ = 0.69, *p* = 0.006) [52]. Additionally, preoperative apparent diffusion coefficient (ADC) values correlated with postoperative outcomes (ρ = −0.58, *p* = 0.027) [52]. Furthermore, following stereotactic evacuation of small basal ganglia hemorrhages, Liao et al. demonstrated that higher FA and better CST preservation at one month were associated with higher Fugl–Meyer scores and lower modified Rankin Scale scores at six months compared to patients receiving medical treatment [42]. This suggests that early surgical decompression may provide measurable microstructural benefits detectable by diffusion tensor imaging (DTI) [42].

#### 3.5.3. Supplementary Motor Area Syndrome: Interhemispheric DTI Metrics and Recovery

Oda et al. (2018) studied 11 brain tumor patients with postoperative SMA syndrome, all recovering within 5–30 days [50]. Using DTI tractography, they extracted interhemispheric fibers “between contralateral SMA and ipsilateral primary motor cortex” and defined NFidx as the “number of tracts” connecting these regions. NFidx was higher in the early recovery group (8923.16 ± 1512.04 (early) vs. 4726.40 ± 1789.46 (late), *p* = 0.002). Tumor volume differed between groups (18.69 ± 12.11 cm^3^ vs. 47.10 ± 26.83 cm ^3^ in the early and late groups, respectively (*p* = 0.028). NFidx > 8000 occurred in 6/7 early recovery and 0/4 late-recovery patients; NFidx < 8000 occurred in 1/7 early recovery and 4/4 late-recovery patients (Fisher exact *p* = 0.0152). The authors noted the limitations of DTI tractography in tumor settings and described their results as preliminary evidence for DTT-derived indices predicting SMA syndrome recovery [50].

Tuncer et al. (2023) studied 56 patients; 39/56 (69.6%) developed acute SMA syndrome, recovering by 3 months, while 30/56 (54%) showed persistent fine motor deficits at 3 months [34]. They computed tract “disconnection severity” using the HCP842 tractography atlas, defined as the percentage of streamlines intersecting the lesion mask. Robust regression showed that SMA–SMA fiber disconnection severity through the central corpus callosum correlated with longer acute symptom duration (unadjusted β = 1.73, robust SE 0.65, *p* = 0.01). LASSO logistic regression found M1–M1 fiber disconnection in midposterior corpus callosum associated with persistent deficits (OR 2.02, 95% CI 1.12–3.6, *p* = 0.02). They noted the limitations of atlas-based disconnection estimates, which may not fully represent individual tract anatomy variability [34].

### 3.6. Language Outcomes

In the reviewed literature, language-related endpoints were investigated through (i) cohort studies of tumor surgery, primarily glioma, utilizing tract-specific diffusion metrics and/or tractography-derived indices [17,19,35,45,49], and (ii) cohort studies of anterior temporal lobe resection (ATL) for temporal lobe epilepsy (TLE) employing voxelwise diffusion analyses correlated with language performance [43,46]. The most frequently examined pathways included the dorsal (arcuate fasciculus and closely related superior longitudinal fasciculus components) and ventral (inferior fronto-occipital, inferior longitudinal, and uncinate fasciculi) streams. The outcomes assessed encompassed global aphasia measures (e.g., the Western Aphasia Battery), naming, fluency, and sentence repetition. A detailed overview of the language-related neuropsychological instruments (e.g., naming, fluency, aphasia batteries), assessment timepoints, and study-specific outcomes is provided in Supplementary Table S1.

#### 3.6.1. Dorsal Stream Biomarkers (Arcuate Fasciculus/SLF)

Kinoshita et al. (2014) conducted a study on preoperative tensor-metric prediction of postoperative language recovery in 12 right-handed patients with left-hemisphere supratentorial tumors [49]. This study quantified fractional anisotropy (FA) in the arcuate fasciculus (AF), including a relative FA metric, and evaluated language abilities using the Western Aphasia Battery (WAB) preoperatively and postoperatively. Results showed a positive correlation between preoperative relative AF FA and postoperative improvement in the total WAB score (r = 0.77, *p* = 0.0056), with significant associations in naming (*p* = 0.018), reading (*p* = 0.029), and writing (*p* = 0.012). Using K-means clustering, patients were categorized into high relative FA (n = 4) and low relative FA (n = 8) groups, with all patients in the high FA group showing improvement, while improvement in the low FA group was less consistent (4/8) [49].

Caverzasi et al. (2016) evaluated language outcomes following glioma surgery using preoperative diffusion imaging and postoperative assessments [17]. Tract status was categorized using an altered fiber tractography density (AFTD) framework and dichotomized into “preserved” versus “affected” for predictive modeling. The arcuate fasciculus (AF) and temporoparietal component of the superior longitudinal fasciculus (SLF-tp) are the key dorsal pathways associated with language deficits. In patients without deficits, AF and SLF-tp were preserved in all cases. In patients with high-grade gliomas and language deficits at discharge, a logistic regression model using AF/SLF-tp tract status predicted persistent deficits at follow-up (*p* = 0.005), with a sensitivity and positive predictive value of 86% and a specificity and negative predictive value of 75% [17].

Single-case neurosurgical evidence shows the role of dorsal stream tractography in tracking the evolution of postoperative language. Chernoff et al. (2020) documented a case of a 26-year-old right-handed male with left inferior parietal glioma infiltrating the arcuate fasciculus (AF), who underwent awake craniotomy [19]. Probabilistic tractography was used to quantify AF integrity compared to healthy controls using leave-one-out masking and normalized streamline counts. Postoperatively, the left AF streamline count decreased to 106, four standard deviations below the control mean (t51 = 3.79, *p* < 0.0004), while right-hemisphere counts remained within one standard deviation. The patient experienced severe postoperative aphasia that resolved within a week, leaving her with sentence repetition impairment. The sentence repetition accuracy was 94% preoperatively, 72.2% at 1 month, and 90% at 3.5 months, with a decrease at 1 month and an increase at 3.5 months. Methodological caveats included streamline count interpretation issues related to acquisition choices and confounding factors, such as edema [19].

#### 3.6.2. Ventral Stream Biomarkers (IFOF/ILF/UF)

Tomasino et al. (2024) provided extensive cohort evidence linking diffusion metrics to postoperative language performance in 30 patients with left temporoinsular diffuse low-grade gliomas [45]. Language assessments were conducted preoperatively, one week postoperatively, and four months postoperatively. The study evaluated inferior fronto-occipital fasciculus (IFOF) microstructural indices (FA, MD, AD, RD) and tractography indices. Postoperatively, many patients showed performance below normative cutoffs on language tasks (56% for noun naming and 64% for verb naming), with some impairments persisting at follow-up (24% and 44%, respectively). Changes in verb naming correlated with left IFOF voxel count (ρ = 0.541, *p* = 0.005) and right IFOF streamlines (ρ = 0.501, *p* = 0.011), as well as right IFOF AD (ρ = 0.631, *p* < 0.001) and MD (ρ = 0.414, *p* = 0.049). At follow-up, verb naming remained associated with left hemisphere indices and bilateral diffusivity metrics. Discriminative analysis showed that early postoperative streamline reduction above 79% identified patients with immediate lexico-semantic disorder (AUC: 0.875, sensitivity: 88.9%, specificity: 81.2%). The study found left-right IFOF asymmetries and differences based on radiological patterns, although tract-based measures depended on modeling choices [45].

Evidence regarding the inferior longitudinal fasciculus (ILF) and its impact on naming outcomes has been predominantly obtained from neurosurgical observations. Shinoura et al. (2010) documented a case in which diffusion tensor imaging (DTI) fiber tracking indicated an interruption of the left ILF, which was associated with a significant postoperative decline in object naming ability (with scores decreasing from 80 to 27), while other language functions remained unaffected [30]. In the same study, longitudinal diffusion/tractography analysis of the ILF revealed a reduction in tract extent, quantified as a 35.4% decrease in the number of voxels within the tract, along with alterations in diffusion-derived properties, specifically increases in mean fractional anisotropy (FA) and axial diffusivity (AD) postoperatively for the remaining tract voxels [30].

#### 3.6.3. Diffusion Correlates of Fluency and Naming

Two cohort studies examining anterior temporal lobectomy (ATL) for temporal lobe epilepsy (TLE) linked postoperative language performance to diffusion tensor metrics using voxel-wise methods. Yogarajah et al. (2010) studied 46 ATL patients (26 left, 20 right) with pre-and postoperative diffusion imaging, using tract-based spatial statistics to identify diffusion changes and correlate them with neuropsychological assessments [46]. In the left ATL cohort, they found an increased fractional anisotropy (FA) of 8% and parallel diffusivity of 6% in contralateral pathways, including the internal/external capsule and superior longitudinal fasciculus/corona radiata. These increases correlated with verbal fluency (FA: r = 0.48, *p* = 0.009; parallel diffusivity: r = 0.47, *p* = 0.009) and object naming (FA: r = 0.46, *p* = 0.027; parallel diffusivity: r = 0.52, *p* = 0.008) scores. The authors noted that postoperative diffusion changes cannot be attributed to a single biological process [46].

Pustina et al. (2014) studied diffusion and language outcomes in 26 anterior temporal lobectomy patients (11 left TLE, 15 right TLE) versus controls [43]. Diffusion data were acquired at 3T with 32 directions (b = 850), analyzing fractional anisotropy (FA) across the preoperative and one-year postoperative periods. In left ATL patients, preoperative phonemic fluency correlated with FA values in postoperative FA-increase clusters in the left superior corona radiata (r = 0.73, *p* = 0.03), right superior longitudinal fasciculus (SLF) (r = 0.70, *p* = 0.02), and right uncinate fasciculus (r = 0.64, *p* = 0.05). Postoperative phonemic and semantic fluency correlated with FA in the right SLF cluster (r = 0.66, *p* = 0.04; r = 0.74, *p* = 0.02). The authors noted that the small sample size limited their ability to explain FA changes [43].

Chernoff et al. (2018) also offered diffusion-based quantification of the frontal aslant tract (FAT) in a glioma case that exhibited changes in speech production, such as a reduced mean length of utterance and altered timing measures [18]. For the left FAT, the study presented pre/post tract diffusion metrics across various tract probability thresholds. While the mean FA showed no significant difference at several thresholds (e.g., threshold 5%: 0.55 ± 0.06 vs. 0.56 ± 0.07; *p* = 0.26), there were significant decreases in mean diffusivity (e.g., 0.99 ± 0.13 vs. 0.90 ± 0.11; *p* = 1.73 × 10^−16^), radial diffusivity (0.66 ± 0.10 vs. 0.57 ± 0.08; *p* = 1.73 × 10^−25^), and axial diffusivity (1.66 ± 0.21 vs. 1.57 ± 0.19; *p* = 2.42 × 10^−7^) at the same threshold [18].

### 3.7. Higher Cognitive Function Outcomes

Three studies assessed higher cognitive functioning in relation to diffusion tensor imaging (DTI)-derived microstructural metrics, primarily focusing on FA and/or MD, across different neurosurgical populations, including glioma resection, moyamoya disease (MMD) revascularization, and shunt surgery for idiopathic normal-pressure hydrocephalus (iNPH). Across these studies, the pathways examined overlapped around fronto-parietal association tracts (SLF) and fronto-temporal/limbic pathways (e.g., uncinate/cingulate–cingulum and callosal regions), although there was considerable heterogeneity in the study design and endpoints.

In the glioma cohort (n = 79), Andreoli et al. extracted the FA and streamline number of 13 bilateral tracts (AF segments, SLF I/II/III, CST, FAT, UF, ILF, IFOF, fornix, and cingulum) and explored tract–cognition relationships using extensive univariate regression modeling, followed by a second model set additionally adjusted for age, given the linear age-related decline in FA and cognition. A consistent pattern in the age-adjusted results showed that FA in long association tracts (particularly the IFOF, ILF, and SLF subcomponents) was linked to memory, visuospatial ability, processing speed, and executive language tasks. In contrast, CST, FAT, UF, and fornix showed no age-adjusted associations with neuropsychological tests in this dataset [37].

In a longitudinal revascularization cohort of adult MMD (n = 25), Kazumata et al. reported the results of multimodal MRI and neuropsychological assessments conducted at baseline and again 2–4 years postoperatively [40]. Postoperative improvement was noted primarily in performance- and speed-related domains, alongside voxel-wise TBSS evidence of postoperative FA increases and MD decreases, including reported changes in the SLF (bilateral FA increases and MD decrease) and FA increases in the left IFOF [40].

Finally, in a prospective iNPH shunt cohort (n = 32; abstract-only), Bubeníková et al. (2025) examined whether DTI metrics tracked clinical improvement after shunt surgery [38]. Within 1 year of shunt placement, improvements in memory and psychomotor speed were reported to correlate with DTI measures in the cingulate gyrus, uncinate fasciculus, superior longitudinal fasciculus (SLF), and corpus callosum, with FA and MD trending toward more physiological values [38].

## 4. Discussion

Across 37 heterogeneous studies spanning tumor, epilepsy, vascular, and hydrocephalus surgeries, quantitative diffusion tensor imaging (DTI) metrics were consistently associated with postoperative neurological deficits and recovery trajectories. A unifying concept across indications is that diffusion-derived measures act as proxies for tract and network “structural reserve”: lower preoperative integrity and greater perioperative disruption generally track with higher functional vulnerability [28,33,37,41,44,47,48,53], whereas longitudinal changes reflect a mixture of injury, decompression, and neuroplastic reorganization [20,22,23,40]. At the same time, the available evidence is dominated by single-center observational cohorts with substantial variation in acquisition, processing, tract definition, and outcome ascertainment, which limits the portability of any single numeric cutoff and supports the interpretation of DTI metrics as risk markers to be integrated with clinical and mapping data rather than as stand-alone decision rules.

### 4.1. Summary of Findings

Motor outcomes provide the largest and most coherent evidence. In cohorts of patients with glioma, hemorrhage, and brainstem surgery, lower preoperative corticospinal tract (CST) fractional anisotropy (FA) (often expressed as an ipsilesional-to-contralesional ratio) and higher diffusivity were repeatedly associated with a higher probability of new postoperative weakness and reduced likelihood of complete recovery. Several studies have additionally reported promising discrimination using tractography-derived indices (e.g., fiber/streamline reduction metrics), with good-to-excellent AUC values in selected cohorts; however, these measures are intrinsically sensitive to tracking parameters and perilesional signal changes [20,21,23,28,41,42,48]. For supplementary motor area (SMA) syndrome, the limited available data suggest that interhemispheric motor connectivity (callosal SMA-SMA and M1-M1 fibers) influences both symptom duration and the persistence of fine motor deficits, aligning with a network-disconnection framework rather than a single-tract model [34,50].

For language, the evidence is smaller and more varied, yet it similarly supports a tract-reserve interpretation: outcomes frequently implicate the dorsal stream (AF/SLF), and across different cohorts and cases, tract integrity/status is associated with postoperative aphasia severity and recovery. Notably, several studies caution that postoperative diffusion changes cannot be solely attributed to a single biological process, which limits mechanistic inference, even when clinical correlations are observed [17,19,49].

For higher cognition, the evidence remains sparse, but the available studies point toward long association and limbic pathways (e.g., SLF, IFOF/ILF, cingulum, and callosal segments), and in some datasets, network-level measures as candidate prognostic targets. This is biologically plausible: unlike motor function, many cognitive outcomes are distributed, and tract-level metrics may need to be contextualized within the connectome organization and preoperative cognitive reserve [59,60,61]. Importantly, the current literature in these domains is insufficient to define which diffusion features add independent value beyond age, lesion burden, and baseline neuropsychological performance [37,40].

### 4.2. Comparison with Stroke Literature

When compared with the stroke DTI biomarker literature, the conceptual parallels are strong (especially for CST-based motor prognostication); however, neurosurgical translation is not straightforward. Neurosurgical cohorts combine chronic, often infiltrative pathology with planned tissue disruption, perioperative edema, resection cavities, and susceptibility artifacts that can bias both tensor metrics and tractography. Moreover, functional mapping and surgical strategy are themselves determinants of outcome, introducing confounding factors [1]. The most transferable lesson from stroke is methodological rather than biological: prognostic utility increases when diffusion features are prespecified, combined with clinical predictors in multivariable models, and tested with external validation and calibration rather than being reported as isolated correlations or center-specific cut-off points [11].

### 4.3. Methodological Factors Explaining Heterogeneity

Several methodological factors likely explain the wide range of reported thresholds and effect sizes. Diffusion acquisition varied markedly (field strength, number of directions, b-values, and availability of distortion-correction data), and preprocessing was inconsistently described. Because perilesional and postoperative tissues are particularly vulnerable to motion, susceptibility, and partial volume effects, differences in correction strategies (eddy current, susceptibility and bias-field correction, and brain extraction) can materially influence FA and diffusivity estimates [1,11,20,37,51,62].

Heterogeneity in tract definition is an equally important source of non-portability of DTI. Many studies have relied on manual or semi-manual region-of-interest placement and deterministic tracking with low FA cutoffs, which can inflate the apparent disruption in edematous or infiltrated tissue [63]. Conversely, aggressive thresholds can prematurely terminate the streamlines and underestimate the residual fibers. Tractography-derived metrics, such as streamline count or tract volume, are intuitive, but they are not direct quantitative measures of axon number and are strongly algorithm- and parameter-dependent [64]. Across studies, relative or within-subject metrics (e.g., hemispheric ratios, along-tract sampling, or change-from-baseline) appear more robust than absolute values and may be better candidates for harmonization than absolute values.

There was considerable clinical and statistical heterogeneity. Outcome definitions varied widely, from simple bedside motor grading to comprehensive neuropsychological assessments, with follow-up periods ranging from discharge to over a year. Many studies were underpowered, relied on univariate testing, and rarely assessed whether DTI features provided additional prognostic value beyond established predictors such as baseline deficit, tumor grade, lesion volume, distance-to-tract, and intraoperative mapping or neurophysiology. External validation was infrequent, increasing the likelihood that some published cutoffs are optimistic and specific to the sample.

For clinical application, it is important to differentiate between (i) voxel-wise/ROI-derived tensor metrics (e.g., FA/MD/RD/AD sampled within ROIs or along-tract) and (ii) tractography-derived indices (e.g., streamline count, tract volume, disconnection measures). The former are generally more comparable across pipelines when ROIs and preprocessing are standardized, whereas the latter are highly sensitive to tractography parameters (such as seeding, stopping criteria, and edema/tumor effects on tracking) and thus require more rigorous standardization [1,11,20,37,51,62].

### 4.4. Strengths and Limitations of the Current Evidence

The main strength of this review is the cross-indication synthesis, which shows that tract- and network-level diffusion features repeatedly map onto clinically meaningful postoperative phenotypes, supporting biological plausibility and clinical relevance. In addition, the literature increasingly includes early postoperative and longitudinal imaging, which is essential for distinguishing pre-existing vulnerability from perioperative changes and recovery-related remodeling.

Despite the existing body of evidence, significant limitations persist, accompanied by a considerable risk of bias. The majority of the studies included were single-center observational cohorts, often retrospective in nature, with a predominant focus on glioma surgery. There is a relative paucity of research concerning epilepsy, vascular, or cerebrospinal fluid disorder populations. This imbalance restricts the generalizability of findings, particularly to epilepsy surgery cohorts and minimally invasive techniques. Furthermore, many studies were small in scale and lacked sufficient statistical power, frequently relying on univariate analyses and inconsistently adjusting (or failing to adjust) for major confounders such as baseline deficit severity, lesion volume/grade, extent of resection, tract–lesion distance, and intraoperative mapping. This raises concerns regarding selection bias, residual confounding, and overly optimistic effect estimates. The imaging acquisition and analysis methodologies were heterogeneous and often inadequately reported, complicating both formal risk-of-bias assessment and publication-bias evaluation, and impeding replication efforts. Collectively, and considering the narrative synthesis necessitated by heterogeneity, our findings should be regarded as hypothesis-generating and clinically suggestive rather than conclusive for patient-level decision-making.

### 4.5. Implications for Clinical Practice and Future Directions

From a clinical standpoint, quantitative DTI can be positioned as a complementary tool with three practical roles. First, preoperative tract integrity metrics (especially CST ratios for motor and AF/IFOF integrity for language) may help stratify functional risk, prioritize patients for awake mapping or more extensive intraoperative monitoring, and frame counseling regarding the likelihood and expected time course of recovery. Second, when new deficits occur, early postoperative diffusion changes, interpreted cautiously and ideally in relation to preoperative baselines and the contralateral hemisphere, may support a differential prognosis between transient perioperative effects and more severe structural injury. Third, longitudinal diffusion measures and connectomics can provide objective markers of structural reorganization that can be integrated with rehabilitation planning, including in epilepsy surgery patients with language or memory concerns.

To move from association to clinically deployable prognostication, future work should prioritize (i) harmonized acquisition with distortion correction and transparent quality control; (ii) standardized, anatomically explicit tract definitions or tractometry pipelines with reproducible parameters; (iii) common outcome sets with prespecified time points (e.g., early postoperative and 3- to 6-month follow-up); (iv) multivariable prediction modeling that tests incremental value over clinical and mapping data; (v) external validation across centers and scanners with calibration and clinical-utility analyses; and (vi) reporting aligned with established prediction-model and imaging-biomarker guidance. Multicenter prospective studies that include epilepsy surgery cohorts and leverage modern diffusion methods (while maintaining compatibility with DTI-derived metrics) will be particularly important for defining which biomarkers are robust enough to inform patient-specific counseling in the future. With these steps, quantitative diffusion metrics can evolve from descriptive adjuncts to reliable components of individualized surgical planning and postoperative care.

## 5. Conclusions

In conclusion, across diverse neurosurgical indications, quantitative DTI provides clinically meaningful markers of white matter integrity that are consistently associated with postoperative motor and language outcomes and show promise for cognitive and gait domains. The strongest and most reproducible evidence supports CST-based FA and diffusivity asymmetry as correlates of postoperative weakness and recovery, while language outcomes appear to depend on the integrity of the dorsal (AF/SLF) and ventral (IFOF/ILF/UF) pathways and on distributed network reorganization after resection, including in ATL cohorts for temporal lobe epilepsy. At present, heterogeneity in acquisition, tract definition, outcome ascertainment, and statistical validation precludes universal thresholds and argues against stand-alone prognostic use. Progress toward clinical translation will require harmonized pipelines, rigorous prospective multicenter validation, and integrated models that combine diffusion features with clinical variables, lesion characteristics, and functional mapping or neurophysiology. Such efforts would clarify the role of diffusion biomarkers in epilepsy and neurosurgical practice, enabling more accurate risk counseling and better-targeted rehabilitation.

## Figures and Tables

**Table 1:** General characteristics of the 37 included studies.

Study (Year)	Condition/Surgery Type	Primary Functional Domain
Gong et al. (2025) [22]	Motor-eloquent gliomas	Motor
Horikawa et al. (2025) [23]	Recurrent glioma patient	Motor
Roth et al. (2025) [44]	Gliomas near CST	Motor
Figueredo et al. (2024) [21]	Primary motor-area gliomas	Motor
Liu et al. (2024) [27]	WHO II/IV gliomas near CST	Motor
Ivren et al. (2023) [25]	Motor-area gliomas	Motor
Tuncer et al. (2023) [34]	SMA gliomas	Motor
Muir et al. (2022) [29]	Motor-eloquent gliomas	Motor
Cepeda et al. (2021) [20]	Gliomas ≤ 2 cm from CST	Motor
Laundre et al. (2005) [26]	Mass lesions incl. glioma	Motor
Khan et al. (2019) [41]	Mixed supratentorial intra-axial tumors	Motor
Oda et al. (2018) [50]	SMA tumors	Motor
Sollmann et al. (2018) [53]	Motor-eloquent gliomas	Motor
Gao et al. (2017) [47]	Gliomas near CST	Motor
Martino et al. (2017) [28]	LGG with long-term paresis	Motor
Sollmann et al. (2017) [33]	Eloquent brain tumors (mixed)	Motor
Ius et al. (2016) [48]	LGGs involving the CST	Motor
Hou et al. (2015) [24]	Gliomas adjacent to PT	Motor
Liao et al. (2025) [42]	Basal ganglia hemorrhage surgery	Motor
Shinoura et al. (2006) [36]	Metastatic brain tumor resection	Motor
Stadlbauer et al. (2007) [51]	Gliomas near CST	Motor
Yao et al. (2015) [52]	Brainstem surgery	Motor
Shinoura et al. (motor) (2006) [36]	Gliomas near M1	Motor
Drane et al. (2014) [39]	Temporal lobe epilepsy surgery	Language
Chernoff et al. (2020) [19]	Left parietal glioma	Language
Tomasino (2024) [45]	Glioma near AF	Language
Chernoff et al. (2018) [18]	Frontal glioma resection	Language
Caverzasi et al. (2016) [17]	Language-eloquent gliomas	Language
Sollmann et al. (language) (2016) [32]	Perisylvian gliomas	Language
Sierpowska et al. (2015) [31]	Left frontal glioma	Language
Pustina et al. (2014) [43]	Adult ATL for TLE	Language
Kinoshitaa et al. (2014) [49]	Language recovery after tumor resection	Language
Shinoura et al. (language) (2009) [30]	Temporal glioma	Language
Yogarajah et al. (2010) [46]	Adult ATL for temporal lobe epilepsy	Language
Andreoli et al. (2023) [37]	Glioma patients	Cognitive
Kazumata et al. (2019) [40]	Adult Moyamoya revascularization	Cognitive
Bubeníková et al. (2025) [38]	Idiopathic NPH shunt surgery	Cognition/gait

## Data Availability

The original contributions presented in this study are included in the article. Further inquiries should be directed to the corresponding author.

## Supplementary Materials

The following supporting information can be downloaded at: https://www.mdpi.com/article/10.3390/life16010115/s1, Table S1: neuropsych_exams.

## Abbreviations

The following abbreviations are used in this manuscript.

AD	Axial diffusivity
ADC	Apparent diffusion coefficient
AF	Arcuate fasciculus
AFTD	Altered fiber tractography density
ATL	Anterior temporal lobectomy
AUC	Area under the curve
CI	Confidence interval
CST	Corticospinal tract
dMRI	Diffusion magnetic resonance imaging
DSI	Diffusion spectrum imaging
DTI	Diffusion tensor imaging
FA	Fractional anisotropy
FAT	Frontal aslant tract
FLAIR	Fluid-attenuated inversion recovery
FSL	FMRIB Software Library
HCP	Human Connectome Project
IFOF	Inferior fronto-occipital fasciculus
ILF	Inferior longitudinal fasciculus
iNPH	Idiopathic normal-pressure hydrocephalus
LGG	Low-grade glioma
MD	Mean diffusivity
MEP	Motor evoked potential
MMD	Moyamoya disease
MRI	Magnetic resonance imaging
mRS	Modified Rankin Scale
NF	Number of fibers
NFidx	Fiber number index
OR	Odds ratio
RD	Radial diffusivity
ROC	Receiver operating characteristic
ROI	Region of interest
rFA	Relative fractional anisotropy
SLF	Superior longitudinal fasciculus
SMA	Supplementary motor area
TBSS	Tract-based spatial statistics
TLE	Temporal lobe epilepsy
UF	Uncinate fasciculus
WAB	Western Aphasia Battery
WHO	World Health Organization

## Appendix A

PubMed search query: (diffusion tensor OR DTI OR tractography OR “fiber tracking” OR “Diffusion Tensor Imaging”) AND (motor OR language OR aphasia OR paresis OR “functional outcome” OR “motor recovery” OR “cortical reorganization” OR plasticity) AND (surgery OR resection OR postoperative OR “post-operative” OR craniotomy).
