# Supplementary material for: Diffusion Tensor Imaging Biomarkers to Predict Neurological Outcomes in Brain Surgery: A Systematic Review
Source: Life (Basel). 2026 Jan 13;16(1):115. doi: 10.3390/life16010115 (PMC12843495; doi:10.3390/life16010115)
Supplement: Supplementary file 1 [file life-16-00115-s001.zip › life-4091793-SI.pdf]

Supplementary Table S1. Neuropsychological assessment batteries and key findings in the included studies。

Study	Sample / surgery	Neuropsychological measures	Assessment timepoints	Key neuropsychological findings	Main diffusion–behavior links reported
Andreoli et al., 2023 [37]	Glioma patients (n=79); diffusion tractography feasibility study with neuropsychological profiling.	RBANS (Total + Immediate Memory, Visuospatial/Constructional, Language, Attention, Delayed Memory); NAB Naming; phonemic fluency (FAS); semantic fluency; WAIS-IV Similarities; Trails A/B; Stroop; Grooved Pegboard; TOPF (premorbid IQ).	Baseline (preoperative).	Variable performance; mean RBANS Total index $z = -0.5 \pm 1.3$ (Immediate Memory −0.6; Delayed Memory −0.6; Language −0.6; Attention −0.5; Visuospatial −0.1).	Age-adjusted tract FA/streamline metrics associated with specific tests (e.g., left IFOF/ILF with list learning; left SLF II with phonemic fluency; left SLF II with Trails A/B).
Caverzasi et al., 2016 [17]	Glioma resection cohort with complete pre/post HARDI tractography (n=35; 14 low-grade, 21 high-grade gliomas).	Clinical language evaluation (by 2 clinicians): spontaneous speech, reading, object/picture naming (64-item panel), comprehension, counting; spelling and graded difficulty items.	Baseline (24–48 h pre-op); discharge (≤3 days); long-term follow-up (>3 months).	Language outcome categories: 23/35 no deficit; 5/35 new deficit at discharge but recovered at follow-up; 4/35 new persistent deficit; 3/35 persistent pre-existing deficit.	Resection/preservation of dorsal and ventral language tracts related to acute deficits and long-term recovery (tract-specific prediction model).
Chernoff et al., 2018 [18]	Two neurosurgical case studies (tumor and epilepsy surgery).	Connected speech elicitation: Cookie Theft picture description (BDAE) with fluency metrics (e.g., mean length of utterance); picture naming (Snodgrass & Vanderwart).	Pre-op and post-op language assessment (case-based).	FAT case: postoperative reduction in speech fluency (shorter mean length of utterance). ILF case: naming accuracy decreased (95%→74%) with slower response times.	Disruption of FAT linked to speech planning/fluency; ILF disruption linked to lexical retrieval/naming.
Chernoff et al., 2020 [19]	Single-case with left arcuate fasciculus resection (parietal glioma).	Comprehensive language battery including sentence repetition, picture/word/number naming, word reading, picture–word matching; praxis tasks; accuracy and RT compared with controls.	Early post-op (3–6 days), 1 week, formal testing at 3 weeks; follow-up at ~1 month and ~3.5 months.	Global aphasia early post-op with later selective repetition deficit. Sentence repetition accuracy: 94% pre-op → 72% at 1 month → 90% at 3.5 months (self-corrections increased at 1 month). Naming largely preserved.	Focal AF resection associated with transient repetition impairment and subsequent language network reorganization/connectivity changes.

Kinoshita et al., 2014 [49]	Left supratentorial tumor resection (n=12; right-handed).	Western Aphasia Battery (WAB): total score + subtests (Naming, Reading, Writing).	Pre-op and early post-op (≤12 days after surgery).	Pre-op relative FA of arcuate fasciculus correlated with postoperative improvement on WAB Total (r=0.77), Naming (r=0.77), Reading (r=0.64), and Writing (r=0.60).	Higher preoperative AF FA predicted better early language recovery.
Tomasino et al., 2024 [45]	Left temporo-insular diffuse low-grade glioma (DLGG) resection in dominant hemisphere; awake surgery with mapping + real-time neuropsych testing; n=30 adults; pre-op 3T DTI.	Fluid intelligence: Raven matrices; Executive: backward digit span (working memory), verbal fluency; Praxis: ideomotor apraxia + oral praxis; Short-term memory: digit span; Language/aphasia battery: token test, auditory comprehension (nouns/verbs), phonological discrimination, noun & verb naming, Pyramids and Palm Trees, auditory & visual lexical decision, reading/writing/repetition (words/pseudowords).	Preoperative (same day as MRI); postoperative 1 week; follow-up 4 months.	Most tasks within normal range pre-op; at 1 week, transient decline mainly in lexico-semantics (% below cut-off: noun naming 56%, verb naming 64%, fluency 50%, token test 36%, visual lexical decisions 63.6%); at 4 months, broad improvement but persistent lexico-semantic deficits in a subset (verb naming 44%, visual lexical decisions 47.8% below cut-off).	Quantitative IFOF indices (NS, NV, V, L, A; FA, MD, AD, RD) showed degraded left vs right IFOF.<br>Left/right IFOF status correlated with immediate post-op decline and with follow-up recovery for naming nouns/verbs, token test, phonological fluency and lexical decisions; ROC: >79% reduction of left IFOF streamlines predicted pathological verb naming at 1 week (AUC 0.875; sens 88.9%, spec 81.2%, accuracy 84%).
Shinoura et al., 2010 [30]	Case report: repeated tumor resection with injury to left ILF.	Standard Language Test of Aphasia (SLTA) domains (auditory comprehension, reading, writing, calculation); object naming using 100 Snodgrass & Vanderwart pictures.	Pre-op and post-op (after second operation).	Selective naming impairment: 80/100 pre-op → 27/100 post-op; other SLTA domains largely unaffected.	Left ILF disruption associated with object naming deficit.
Pustina et al., 2014 [43]	Anterior temporal lobectomy for temporal lobe epilepsy (n=26; 11 left, 15 right) + healthy controls.	Phonemic fluency: Controlled Oral Word Association Test (FAS). Semantic fluency: Animal Naming.	Preoperative and 1-year postoperative neuropsychological testing.	Verbal fluency performance related to pre/post white-matter FA changes in left temporal regions.	Temporal white-matter FA clusters correlated with phonemic and semantic fluency.

Yogarajah et al., 2010 [46]	Anterior temporal lobe resection for temporal lobe epilepsy (n=46).	McKenna Graded Naming Test (30 line drawings); letter fluency (letter "S"); category fluency (animals).	Pre-op and post-op (mean 4.5 months).	Language scores related to postoperative white-matter changes (letter/category fluency and naming).	Letter fluency correlated with FA increases in left superior frontal/deep WM; graded naming correlated with FA increases in left cingulum (in left ATL subgroup).
Stasenko et al., 2023 [54]	Epilepsy surgery cohort with pre/post memory testing and DTI connectomics (left TLE n=21; right TLE n=23).	CVLT-II (verbal learning & long-delay free recall); BVMT-R (visual learning & delayed recall); WTAR (premorbid IQ). Memory decline defined using RCI-PE.	Pre-op and post-op neuropsychological testing (mean 15.9 ± 6.3 months).	Higher rate of postoperative verbal memory decline in left TLE group; network-based measures used to classify decline (reported AUC up to 0.84).	Preoperative structural connectome measures predicted postoperative verbal memory decline in left TLE; limited prediction for right TLE or visual memory decline.
Kazumata et al., 2019 [40]	Adult moyamoya disease undergoing combined revascularization (n=25; follow-up cognitive testing completed by most participants).	WAIS-III (FIQ/VIQ/PIQ + indices); frontal-executive battery (TMT A/B, WCST, Continuous Performance Test, Stroop); Wechsler Memory Scale (WMS-R).	Pre-op and >12 months post-op (mean 21.2 ± 9.1 months).	Post-op improvements reported in WAIS-III FIQ, PIQ, perceptual organization and processing speed; WMS general memory and delayed recall improved.	FA increased (and MD decreased) postoperatively in tracts including SLF; connectivity changes correlated with cognitive improvement.

Abbreviations: AF = arcuate fasciculus; ATL = anterior temporal lobectomy; BDAE = Boston Diagnostic Aphasia Examination; BVMT-R = Brief Visuospatial Memory Test–Revised; COWAT = Controlled Oral Word Association Test; CVLT-II = California Verbal Learning Test–Second Edition; DTI = diffusion tensor imaging; FA = fractional anisotropy; FIQ/PIQ/VIQ = full-scale/performance/verbal intelligence quotient; HARDI = high angular resolution diffusion imaging; IFOF = inferior fronto-occipital fasciculus; ILF = inferior longitudinal fasciculus; RBANS = Repeatable Battery for the Assessment of Neuropsychological Status; RCI-PE = reliable change index with practice effect correction; SLF = superior longitudinal fasciculus; SLTA = Standard Language Test of Aphasia; TLE = temporal lobe epilepsy; TMT = Trail-Making Test; UF = uncinate fasciculus; WAB = Western Aphasia Battery; WAIS = Wechsler Adult Intelligence Scale; WCST = Wisconsin Card Sorting Test; WMS-R = Wechsler Memory Scale–Revised; WTAR = Wechsler Test of Adult Reading.
